# Evaluating Hierarchical Structure in Music Annotations

**DOI:** 10.3389/fpsyg.2017.01337

**Published:** 2017-08-03

**Authors:** Brian McFee, Oriol Nieto, Morwaread M. Farbood, Juan Pablo Bello

**Affiliations:** ^1^Center for Data Science, New York University New York, NY, United States; ^2^Music and Audio Research Laboratory, Department of Music and Performing Arts Professions, New York University New York, NY, United States; ^3^Pandora, Inc. Oakland, CA, United States

**Keywords:** music structure, hierarchy, evaluation, inter-annotator agreement

## Abstract

Music exhibits structure at multiple scales, ranging from motifs to large-scale functional components. When inferring the structure of a piece, different listeners may attend to different temporal scales, which can result in disagreements when they describe the same piece. In the field of music informatics research (MIR), it is common to use corpora annotated with structural boundaries at different levels. By quantifying disagreements between multiple annotators, previous research has yielded several insights relevant to the study of music cognition. First, annotators tend to agree when structural boundaries are ambiguous. Second, this ambiguity seems to depend on musical features, time scale, and genre. Furthermore, it is possible to tune current annotation evaluation metrics to better align with these perceptual differences. However, previous work has not directly analyzed the effects of hierarchical structure because the existing methods for comparing structural annotations are designed for “flat” descriptions, and do not readily generalize to hierarchical annotations. In this paper, we extend and generalize previous work on the evaluation of hierarchical descriptions of musical structure. We derive an evaluation metric which can compare hierarchical annotations holistically across multiple levels. sing this metric, we investigate inter-annotator agreement on the multilevel annotations of two different music corpora, investigate the influence of acoustic properties on hierarchical annotations, and evaluate existing hierarchical segmentation algorithms against the distribution of inter-annotator agreement.

## 1. Introduction

Music is a highly structured information medium, containing sounds organized both synchronously and sequentially according to attributes such as pitch, rhythm, and timbre. This organization of sound gives rise to various musical notions of harmony, melody, style, and form. These complex structures include multiple, inter-dependent levels of information that are hierarchically organized: from individual notes and chords at the lowest levels, to measures, motives and phrases at intermediate levels, to sectional parts at the top of the hierarchy (Lerdahl and Jackendoff, 1983). This rich and intricate pattern of structures is one of the distinguishing characteristics of music when compared to other auditory phenomena, such as speech and environmental sound.

The perception of structure is fundamental to how listeners experience and interpret music. Form-bearing cues such as melody, harmony, timbre, and texture (McAdams, 1989) can be interpreted in the context of both short and long-term memory. Hierarchies are considered a fundamental aspect of structure perception, as musical structures are best retained by listeners when they form hierarchical patterns (Deutsch and Feroe, 1981). Lerdahl (1988) goes so far as to advocate that hierarchical structure is absolutely essential for listener appreciation of music since it would be impossible to make associations between nonadjacent segments without it. Hierarchical structure is also experienced by listeners over a wide range of timescales on the order of seconds to minutes in length (Farbood et al., 2015). Although interpretation of hierarchical structure is certainly influenced by acculturation and style familiarity (Barwick, 1989; Clayton, 1997; Drake, 1998; Drake and El Heni, 2003; Bharucha et al., 2006; Nan et al., 2006), there are aspects of it that are universal. For example, listeners group together some elements of music based on Gestalt theory (Deutsch, 1999; Trehub and Hannon, 2006), and infants have been shown to differentiate between correctly and incorrectly segmented Mozart sonatas (Krumhansl and Jusczyk, 1990).^1^

The importance of hierarchical structure in music is further highlighted by research showing how perception of structure is an essential aspect of musical performance (Cook, 2003). Examination of timing variations in performances has shown that the lengthening of phrase endings corresponds to the hierarchical depth of the ending (Todd, 1985; Shaffer and Todd, 1987). Performers also differ in their interpretations much like listeners (or annotators) differ in how they perceive structure. A combination of converging factors can result in a clear structural boundary, while lack of alignment can lead to an ambiguous boundary. In ambiguous cases, listeners and performers may focus on different cues to segment the music. This ambiguity has not been the focus of empirical work, if only because it is (by definition) hard to generalize.

Unsurprisingly, structure analysis has been an important area of focus for music informatics research (MIR), dealing with tasks such as motif-finding, summarization and audio thumbnailing, and more commonly, segmentation into high-level sections (see Paulus et al., 2010 for a review). Applications vary widely, from the analysis of a variety of musical styles such as jazz (Balke et al., 2016) and opera (Weiß et al., 2016), to algorithmic composition (Herremans and Chew, 2016; Roy et al., 2016) and the creation of mash-ups and remixes (Davies et al., 2014).

This line of work, however, is often limited by two significant shortcomings. First, most existing approaches fail to account for hierarchical organization in music, and characterize structure simply as a sequence of non-overlapping segments. Barring a few exceptions (McFee and Ellis, 2014a,b; McFee et al., 2015a; Grill and Schlüter, 2015), this flat temporal partitioning approach is the dominant paradigm for both the design and evaluation of automated methods. Second, and more fundamentally, automated methods are typically trained and evaluated using a single “ground-truth” annotation for each recording, which relies on the unrealistic assumption that there is a single valid interpretation to the structure of a given recording or piece. However, it is well known that perception of musical structure is ambiguous, and that annotators often disagree in their interpretations. For example, Nieto (2015) and Nieto et al. (2014) provide quantitative evidence of inter-annotator disagreement, differentiating between content with high and low ambiguity, and showing listener preference for over- rather than under-segmentation. The work of Bruderer (2008) shows that annotators tend to agree when quantifying the degree of ambiguity of music segment boundaries, while in Smith et al. (2014) disagreements depend on musical attributes, genre, and (notably) time-scale. Differences in time-scale are particularly problematic when hierarchical structures are not considered, as mentioned above. This issue can potentially result in a lack of differentiation between *superficial* disagreements, arising from different but compatible analyses of a piece, from *fundamental* discrepancies in interpretation, e.g., due to attention to different acoustic cues, prior experience, cultural influences on the listener, etc.

The main contribution of this article is a novel method for measuring agreement between hierarchical music segmentations, which we denote as the *L-measure*. The proposed approach can be used to compare hierarchies of different depths, including flat segmentations, as well as hierarchies that are not aligned in depth, i.e., segments are assigned to the same hierarchical level but correspond to different time-scales. By being invariant to superficial disagreements of scale, this technique can be used to identify true divergence of interpretation, and thus help in isolating the factors that contribute to such differences without being confounded by depth alignment errors.

The L-measure applies equally to annotated and automatically estimated hierarchical structures, and is therefore helpful to both music cognition researchers studying inter-subject agreement and to music informatics researchers seeking to train and benchmark their algorithms. To this end, we also describe three experimental studies that make use of the proposed method. The first experiment compares the L-measure against a number of standard flat metrics for the task of quantifying inter-annotator agreement, and seeks to highlight the properties of this technique and the shortcomings of existing approaches. The second experiment uses the L-measure to identify fundamental disagreements and then seeks to explain some of those differences in terms of the annotators focus on specific acoustic attributes. The third experiment evaluates the performance of hierarchical segmentation algorithms using the L-measure and advances a novel methodology for MIR evaluation that steps away from the “ground-truth” paradigm and embraces the possibility of multiple valid interpretations.

## 2. Corpora

In our experiments, we use publicly available sets of hierarchical structural annotations produced by at least two music experts per track. To the best of our knowledge, the only published data sets that meet these criteria are SALAMI (Smith et al., 2011) and SPAM (Nieto and Bello, 2016).

### 2.1. SALAMI

The publicly available portion of the *Structural Annotations for Large Amounts of Music Information* (SALAMI) set contains two hierarchical annotations for 1,359 tracks, 884 of which have annotations from two distinct annotators and are included in this study. These manual annotations were produced by a total of 10 different music experts across the entire set, and contain three levels of segmentations per track: *fine, coarse*, and *function*. The *fine* level typically corresponds to short phrases (described by lower-case letters), while the *coarse* section represents larger sections (described by upper-case letters). The *function* level applies semantic labels to large sections, e.g., “verse” or “chorus” (Smith et al., 2011). The boundaries of the function level often coincide with those of the coarse level, but for simplicity and consistency with SPAM (described below), we do not use the function level. The SALAMI dataset includes music from a variety of styles, including jazz, blues, classical, western pop and rock, and non-western (“world”) music. We manually edited 171 of the annotations to correct formatting errors and enforce consistency with the annotation guide.^2^ The corrected data is available online.^3^

### 2.2. SPAM

The *Structural Poly Annotations of Music* is a collection of hierarchical annotations for 50 tracks of music, each annotated by five experts. Annotations contain *coarse* and *fine* levels of segmentation, following the same guidelines used in SALAMI. The music in the SPAM collection includes examples from the same styles as SALAMI. The tracks were automatically sampled from a larger collection based on the degree of segment boundary agreement among a set of estimations produced by different algorithms (Nieto and Bello, 2016). Forty-five of these tracks are particularly challenging for current automatic segmentation algorithms, while the other five are more straightforward in terms of boundary detection. In the current work we treat all tracks equally and use all 10 pairs of comparisons between different annotators per track. The SPAM collection includes some of the same audio examples as the SALAMI collection described above, but the annotators are distinct, so annotation data is shared between the two collections.

## 3. Methods for comparing annotations

The primary technical contribution of this work is a new way of comparing structural annotations of music that span multiple levels of analysis. In this section, we formalize the problem statement and describe the design of the experiments in which we test the method.

### 3.1. Comparing flat segmentations

Formally, a *segmentation* of a musical recording is defined by a temporal partitioning of the recording into a sequence of labeled time intervals, which are denoted as *segments*. For a recording of duration *T* samples, a segmentation can be encoded as mapping of samples *t* ∈ [*T*] = {1, 2, …, *T*} to some set of segment labels *Y* = {*y*_1_, *y*_2_, …, *y*_*k*_}, which we will generally denote as a function *S* : [*T*] → *Y*.^4^ For example, *Y* may consist of functional labels, such as *intro* and *verse*, or section identifiers such as *A* and *B*. A *segment boundary* is any time instant at the boundary between two segments. Usually this corresponds to a change of label *S*(*t*) ≠ *S*(*t* − 1) (for *t* > 1), though boundaries between similarly labeled segments can also occur, e.g., when a piece has an *AA* form, or a verse repeats twice in succession.

When comparing two segmentations—denoted as the *reference* *S*^R^ and *estimate* *S*^E^—a variety of metrics have been proposed, measuring either the agreement of segment boundaries, or agreement between segment labels. Two segmentations need not share the same label set *Y*, since different annotators may not use labels consistently, so evaluation criteria need to be invariant with respect to the choice of segment labels, and instead focus on the patterns of label agreement shared between annotations. Of the label agreement metrics, the two most commonly used are *pairwise classification* (Levy and Sandler, 2008) and *normalized conditional entropy* (Lukashevich, 2008).

#### 3.1.1. Pairwise classification

The pairwise classification metrics are derived by computing the set *A* of pairs of similarly labeled distinct time instants (*u, v*) within a segmentation:

(1) A(S):={(u,v)|S(u)=S(v)}.

Pairwise precision (P-Rrecision) and recall (P-Recall) scores are then derived by comparing *A*(*S*^R^) to *A*(*S*^E^):

(2) $$\text{P-Precision}\left(S^{\text{R}},S^{\text{E}}\right):=\frac{\left|A\left(S^{\text{R}}\right)\cap A\left(S^{\text{E}}\right)\right|}{\left|A\left(S^{\text{E}}\right)\right|}$$

(3) $$\text{P-Recall}\left(S^{\text{R}},S^{\text{E}}\right)\;:=\frac{\left|A\left(S^{\text{R}}\right)\cap A\left(S^{\text{E}}\right)\right|}{\left|A\left(S^{\text{R}}\right)\right|}.$$

The precision score measures the correctness of the predicted label agreements, while the recall score measures how many of the reference label agreements were found in the estimate. Because these scores are defined in terms of exact label agreement between time instants, they are sensitive to matching the exact level of specificity in the analysis encoded by the two annotations in question. If *S*^E^ is at a higher (coarser) or lower (finer) level of specificity than *S*^R^, the pairwise scores can be small, even if the segmentations are mutually consistent. Examples of this phenomenon are provided later in Section 4.

#### 3.1.2. Normalized conditional entropy

The normalized conditional entropy (NCE) metrics take a different approach to measuring similarity between annotations. Given the two flat segmentations *S*^R^ and *S*^E^, a joint probability distribution **P**[*y*^R^, *y*^E^] is estimated as the frequency of time instants *t* that receive label *y*^R^ in the reference *S*^R^ and *y*^E^ in the estimate *S*^E^:

(4) $$P\left[y^{\text{R}},y^{\text{E}}\right]\propto \left|\left\{t|\;S^{\text{R}}(t)=y^{\text{R}}\wedge S^{\text{E}}(t)=y^{\text{E}}\right\}\right|$$

From the joint distribution **P**, the conditional entropy is computed between the marginal distributions **P**^R^ and **P**^E^:

(5) $$ℍ\left(P^{\text{E}}|\;P^{\text{R}}\right)=\sum_{y^{\text{R}},y^{\text{E}}}P\left[y^{\text{R}},y^{\text{E}}\right]\log\frac{P^{\text{R}}\left[y^{\text{R}}\right]}{P\left[y^{\text{R}},y^{\text{E}}\right]}$$

The conditional entropy therefore measures how much information the reference distribution **P**^R^ conveys about the estimate distribution **P**^E^: if this value is small, then the segmentations are similar, and if it is large, they are dissimilar.

The conditional entropy is then normalized by log |*Y*^E^|: the maximum possible entropy for a distribution over labels *Y*^E^.^5^ The normalized entropy is subtracted from 1 to produce the so-called *over-segmentation score* NCE_*o*_, and reversing the roles of the reference and estimate yields the *under-segmentation score* NCE_*u*_:

(6) $${\text{NCE}}_{o}\;:=1-\frac{ℍ\left(P^{\text{E}}|P^{\text{R}}\;\right)}{\log\left|Y^{\text{E}}\right|}$$

(7) $${\text{NCE}}_{u}\;:=1-\frac{ℍ\left(P^{\text{R}}\;|P^{\text{E}}\;\right)}{\log\left|Y^{\text{R}}\right|}.$$

The naming of these metrics derives from their application in evaluating automatic segmentation algorithms. If the estimate has large conditional entropy given the reference, then it is said to be *over-segmented* since it is difficult to predict the estimated segment label from the reference: this leads to a small NCE_*o*_. Similar reasoning applies to NCE_*u*_: if ℍ(**P**^R^|**P**^E^) is large, then it is difficult to predict the reference from the estimate, so the estimate is thought to be *under-segmented* (and hence a small NCE_*u*_ score). If both NCE_*o*_ and NCE_*u*_ are large, then the estimate is neither over- nor under-segmented with respect to the reference.

#### 3.1.3. Comparing annotations

When comparing two annotations in which there is no privileged “reference” status for either—such as the case with segmentations produced by two different annotators of equal status—the notions of precision and recall, or over- and under-segmentation can be dubious since neither annotation is assumed to be “correct” or *ground truth*. Arbitrarily deciding that one annotation was the reference and the other was the estimate would produce precision and recall scores, but reversing the roles of the annotations would exchange the roles of precision and recall, since P-Precision(*S*^1^, *S*^2^) = P-Recall(*S*^2^, *S*^1^).

A common solution to this ambiguity is to combine precision and recall scores into a single summary number. This is most often done by taking the harmonic mean of precision *P* and recall *R*, to produce the *F*1-score or *F*-measure:

(8) $$F\;:=2\frac{P\cdot R}{P+R}.$$

For the remainder of this article, we summarize the agreement between two annotations by the *F*-measure, using precision and recall for pairwise classification, and over- and under-segmentation for NCE metrics.

### 3.2. Hierarchical segmentation

A *hierarchical segmentation* is a sequence of segmentations

(9) $$H=(S_{0},S_{1},S_{2},\ldots ,S_{m}),$$

where the ordering typically encodes a coarse-to-fine analysis of the recording. Each *S*_*i*_ in a hierarchy is denoted as a *level*. We assume that the first level *S*_0_ always consists of a single segment which spans the entire track duration.^6^

Most often, when presented with two hierarchical segmentations *H*^R^ and *H*^E^, practitioners assume that the hierarchies span the same set of levels, and compare the hierarchies level-by-level: $S_{1}^{\text{R}}$ to $S_{1}^{\text{E}}$, $S_{2}^{\text{R}},S_{2}^{\text{E}}$, *etc*., or between all pairs of levels (Smith et al., 2011). This results in a set of independently calculated scores for the set of levels, rather than a score that summarizes the agreement between the two hierarchies. Moreover, this approach does not readily extend to hierarchies of differing depths, and is not robust to depth alignment errors, where one annotator's *S*_1_ may correspond to the other's *S*_2_.

To the best of our knowledge, no previous work has addressed the problem of holistically comparing two labeled hierarchical segmentations. Our previous work (McFee et al., 2015a) addressed the unlabeled, boundary-detection problem, which can be recovered as a special case of the more general formulation derived in the present work (where each segment receives a unique label).

#### 3.2.1. Hierarchical label agreement

Given a hierarchical segmentation *H* as defined in Equation (9) and time instants *u, v*, define the *meet* of *u* and *v* under *H* as

(10) $$M(u,v\;|\;H)\;:=\text{max }k\text{ such that }S_{k}(u)=S_{k}(v),$$

that is, *M*(*u, v* | *H*) is the deepest level of *H* where *u* and *v* receive the same label. The meet induces a partial ordering over pairs of time instants: large values of *M*(*u, v* | *H*) indicate a high degree of similarity, and small values indicate low similarity.

To compare two hierarchical segmentations *H*^R^ and *H*^E^, we examine triples of distinct time instants *t, u, v* in terms of the pairwise meets *M*(*t, u* | *H*^R^) and *M*(*t, v* | *H*^R^). We define the reference comparison set for a hierarchy *H* as

(11) A(H) :={(t,u,v) |M(t,u |H)>M(t,v |H )},

that is, the set of triples where (*t, u*) agree at a deeper level than the pair (*t, v*).

Level-independent precision and recall scores—*L-Precision* and *L-Recall*—can be defined, just as in the pairwise classification method of Section 3.1.1, by comparing the size of the intersection to the reference comparison set:

(12) $$\text{L-Precision}\left(H^{\text{R}},H^{\text{E}}\right)\;:=\frac{\left|A(H^{\text{R}})\cap A(H^{\text{E}})\right|}{\left|A(H^{\text{E}})\right|}$$

(13) $$\text{L-Recall}\left(H^{\text{R}},H^{\text{E}}\right)\;:=\frac{\left|A(H^{\text{R}})\cap A(H^{\text{E}})\right|}{\left|A(H^{\text{R}})\right|}.$$

These scores capture the rank-ordering of pairwise similarity between time instants, and can be interpreted as a relaxation of the pairwise classification metrics. We define the *L-Measure* as the harmonic mean of L-Precision and L-Recall.

**Figure 1:**
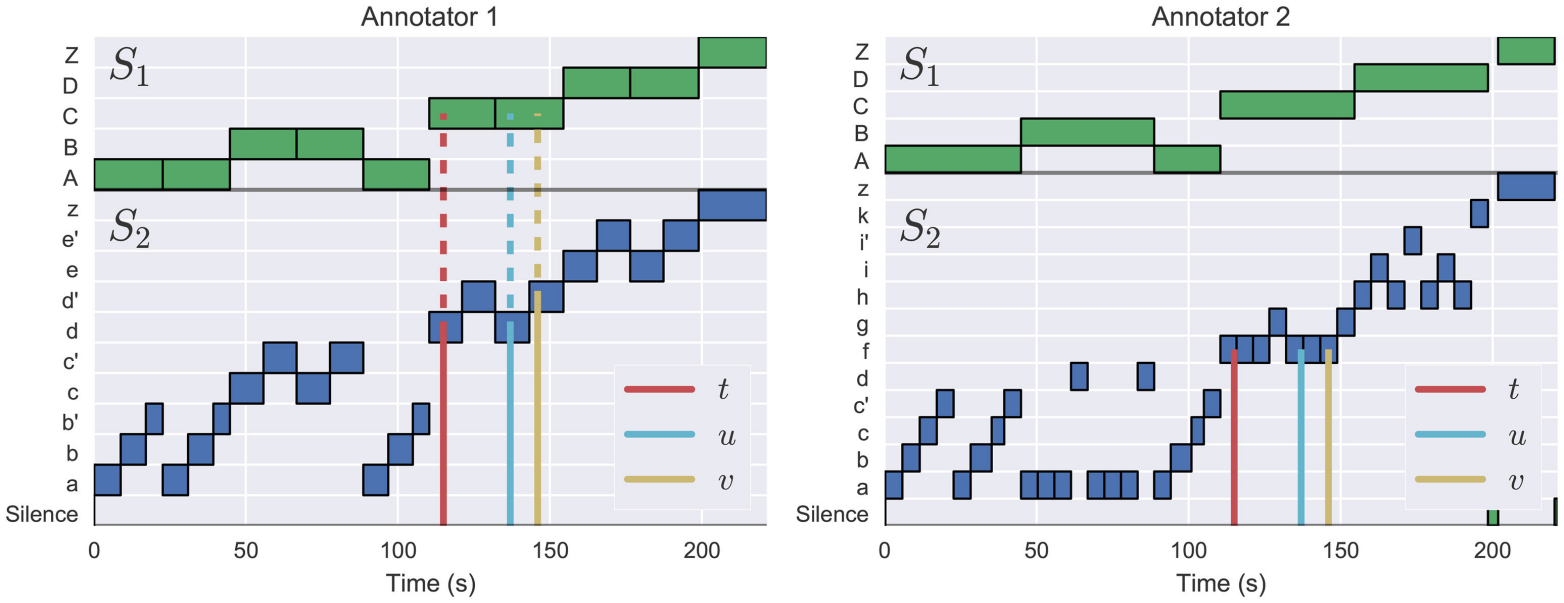
The L-measure is computed by identifying triples of time instants (*t, u, v*) where (*t, u*) meet at a deeper level of the hierarchy (indicated by solid lines) than (*t, v*) (dashed lines), as illustrated in the left plot (Annotator 1). In this example, the left annotation has *M*(*t, u*) = 2 (both belong to lower-level segments labeled as *d*), and *M*(*t, v*) = 1 (both belong to upper-level segments labeled as *C*). The right annotation has *M*(*t, u*) = *M*(*t, v*) = 2: all three instants belong to segment label *f*, as indicated by the solid lines. This triple is therefore counted as evidence of disagreement between the two hierarchies.

Rather than asking if an annotation describes two instants (*u, v*) as the *same* or *different*, the scores defined here ask whether (*t, u*) as *more similar* or *less similar* to each-other than the pair (*t, v*), and whether that ordering is respected in both annotations. An example of this process is illustrated in Figure 1. Consequently, the proposed scores are robust to depth alignment errors between annotations, and readily support comparison between hierarchies of differing depth.

## 4. Experiment 1: L-measures and flat metrics

Our first experiment investigates how the L-measure described above quantifies inter-annotator agreement for hierarchical music segmentation as compared to metrics designed for flat segmentations.^7^

### 4.1. Methods

The data sets described in Section 2 consist of musical recordings, each of which has at least two hierarchical annotations, which are each comprised of flat *upper* (high-level) and *lower* (low-level) segmentations. For each pair of annotations, we compare the L-measure to existing segmentation metrics (pairwise classification and normalized conditional entropy) at both levels of the hierarchy.

From this set of comparisons, we hope to identify examples illustrating the following behaviors: pairs where the flat metrics are small because the two annotations exist at different levels of analysis; and pairs where the flat metrics are large at one level, but small at the other, indicating hierarchical disagreement. In the calculation of all evaluation metrics, segment labels are sampled at a rate of 10 Hz, which is the standard practice for segmentation evaluation (Raffel et al., 2014).

### 4.2. Results and discussion

**Figure 2:**
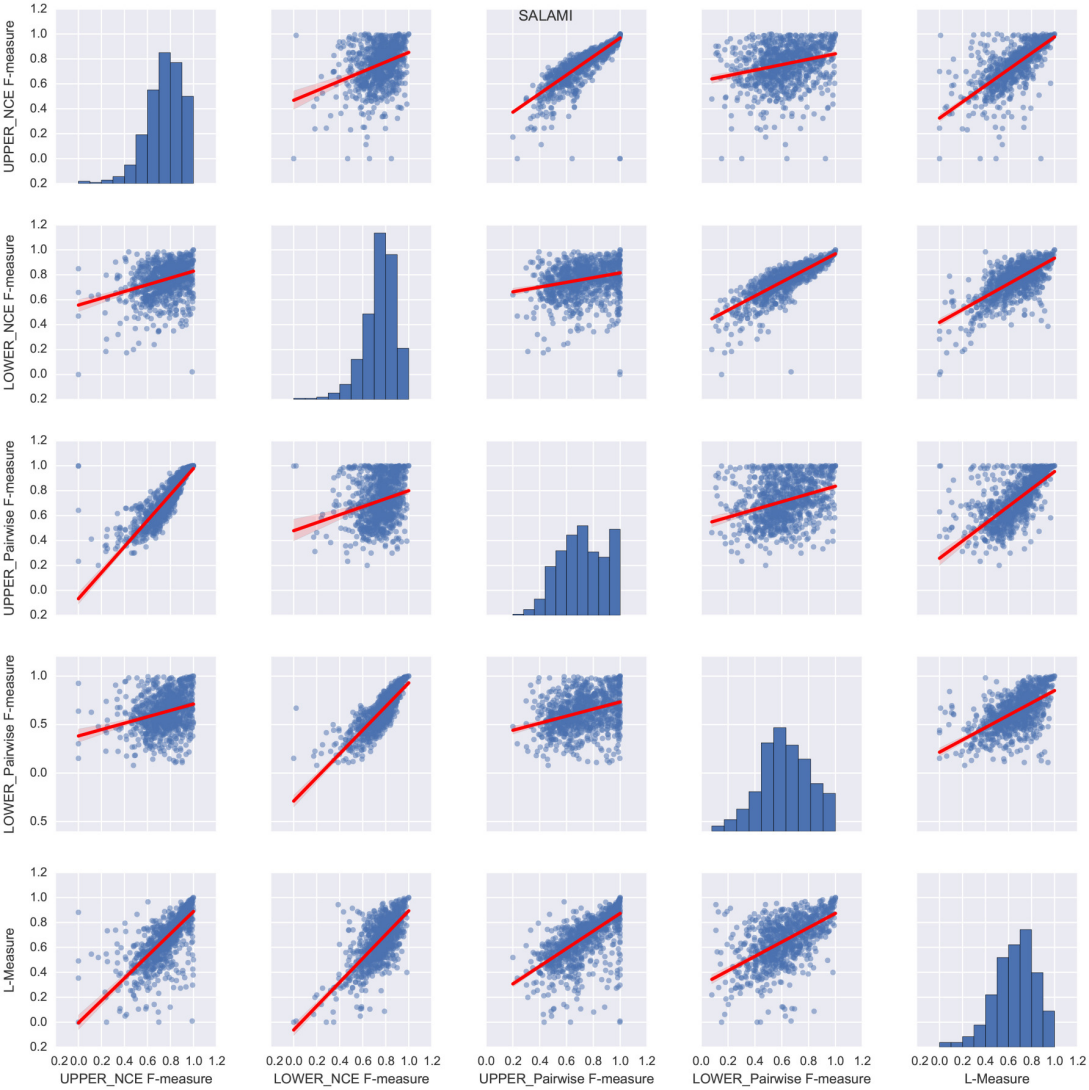
Relations between the different segment labeling metrics on the SALAMI dataset. Each subplot (*i, j*) corresponds to a pair of distinct metrics for *i* ≠ *j*, while the main diagonal illustrates the histogram of scores for the *i*th metric. Each point within a subplot corresponds to a pair of annotations of the same recording. The best-fit linear regression line between each pair of metrics is overlaid in red, with shaded regions indicating the 95% confidence intervals.

Figure 2 illustrates the behavior on SALAMI of the L-measure compared to the flat segmentation metrics (right column), as well as all other pairs of comparisons between metrics. Overlaid in red on each plot is the best-fit robust (Huber's T) linear regression line, with shaded regions indicating the 95% confidence intervals as estimated by bootstrap sampling (*n* = 500 trials). This figure demonstrates a general trend of positive correlation between the L-measure and flat segmentation metrics at both levels, indicating that the L-measure integrates information across the entire hierarchy. Additionally, this plot exhibits a high degree of correlation between the pairwise classification and NCE metrics when confined to a single level. For the remainder of this section, we will focus on comparing L-measure to the pairwise classification metrics, which are more similar in implementation to L-measure.

**Figure 3:**
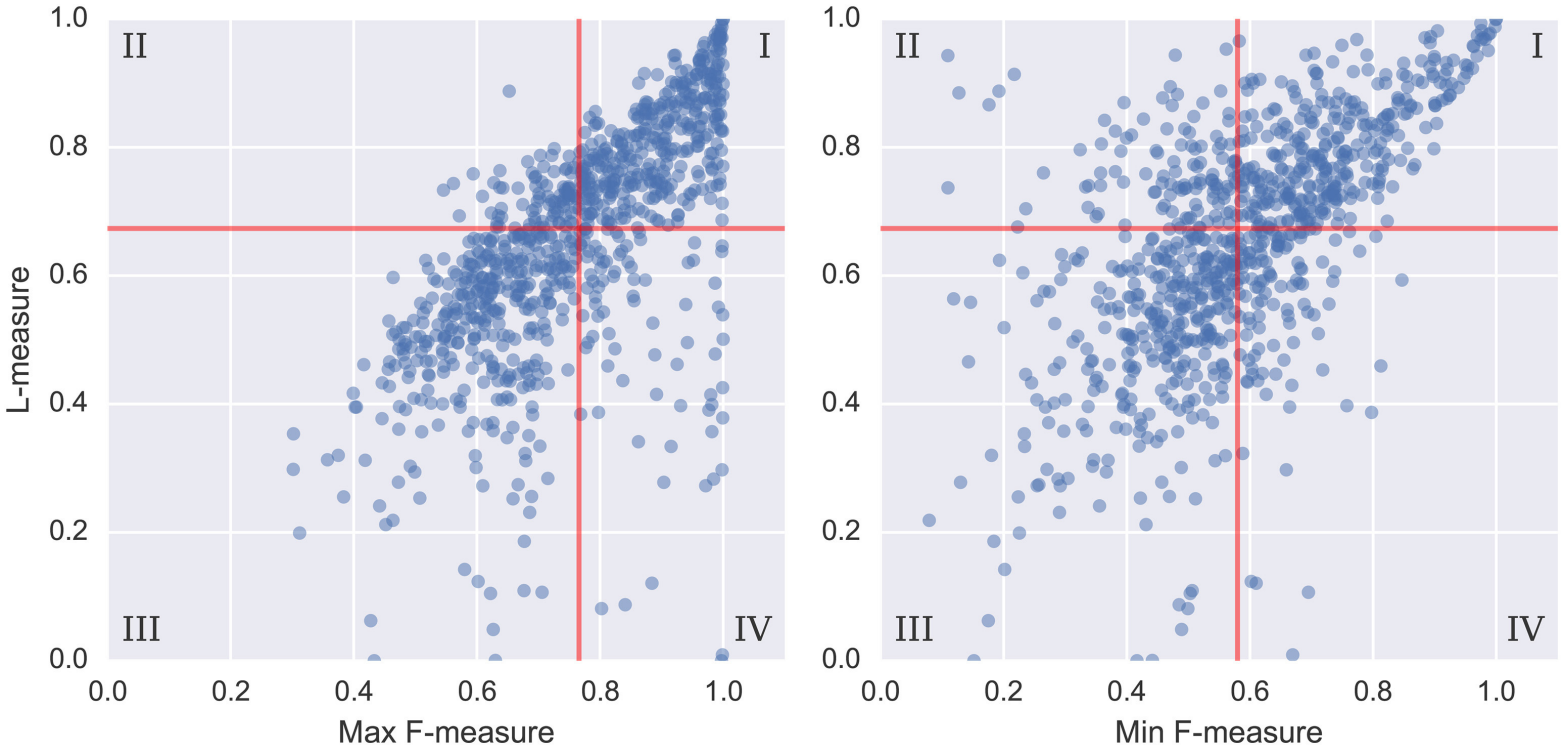
For each pair of annotations in the SALAMI dataset, we compare the L-measure to the maximum and minimum agreement between the upper and lower levels. Agreement is measured by pairwise frame classification metrics. Red lines indicate the median values for each metric. A small maximum F-measure (quadrants II and III in the left plot) indicates disagreement at both levels; a large minimum F-measure (quadrants I and IV in the right plot) indicates agreement at both levels.

To get a better sense of how the L-measure captures agreement over the full hierarchy, Figure 3 compares the L-measure to the maximum and minimum agreements across levels of the hierarchy: that is, *L*(*H*^R^, *H*^E^) compared to $\max\left(F\left(\overset{\text{R}}{\underset{1}{S}},S_{1}^{\text{E}}\right),F\left(S_{2}^{\text{R}},S_{2}^{\text{E}}\right)\right)$. The resulting plots are broken into quadrants I–IV along the median values of each metric, indicated in red. To simplify the presentation, we only compared the L-measure to the pairwise F-measure scores, though the results using normalized conditional entropy scores are qualitatively similar. Of particular interest in these plots are the points where the maximum is small (disagreement at both levels) or the minimum is large (agreement at both levels), and how the L-measure scores these points.

Quantitatively, of the points below the median of maximum F-measure (quadrants II and III of Figure 3, left), 81% lie below the median L-measure (quadrant III). Conversely, the points above the median of minimum F-measure (quadrants I and IV of Figure 3, right) have 75% above the median L-measure (quadrant I). These two quadrants (I and III) correspond to subsets of examples where the L-measure broadly agrees with the pairwise F-measure scores, indicating that there is little additional discriminative information encoded in the hierarchy beyond what is captured by level-wise comparisons. The remaining points correspond to inversions of score from what would be expected by level-by-level comparison: quadrant II in the left plot (9.5% of points), and IV in the right plot (12.6% of points).

**Figure 4:**
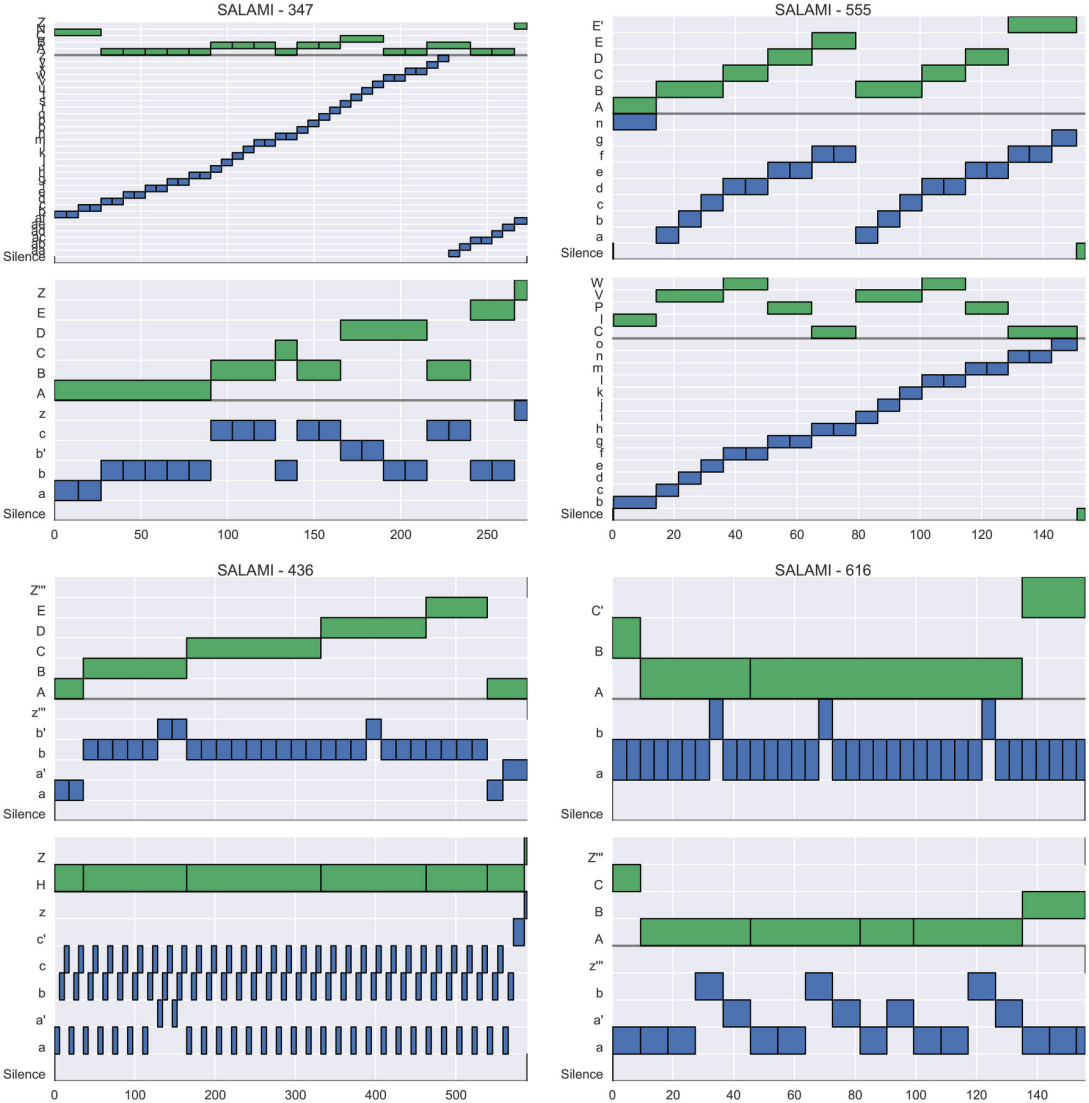
Four example tracks from SALAMI, one drawn from each quadrant of Figure 3 **(Left)**, which compares L-measure to the maximum of upper- and lower-level pairwise F-measure between tracks. For each track, two hierarchical annotations are displayed (top and bottom), and within each hierarchy, the upper level is marked in green and the lower in blue. **(Upper right)** Track 555 (*L* = 0.94, upper *F* = 0.92, lower *F* = 0.69) has high agreement at the upper level, and small agreement at the lower level. **(Upper left)** Track 347 (*L* = 0.89, upper *F* = 0.65, lower *F* = 0.19) has little within-level agreement between annotations, but the upper level of the top annotation is nearly identical to the lower level of the bottom annotation, and the L-measure identifies this consistency. **(Bottom left)** Track 436 (*L* = 0.24, upper *F* = 0.35, lower *F* = 0.44) has little agreement at any level, and receives small scores in all metrics. **(Bottom right)** Track 616 (*L* = 0.30, upper *F* = 0.998, lower *F* = 0.66) has high agreement within the upper level, but disagreement in the lower levels.

Figure 4 illustrates example annotations drawn from each quadrant of the left plot of Figure 3 (across-layer maximum vs. L-measure). The two plots in the left column, corresponding to quadrants II and III, illustrate examples where the flat metrics disagree at both levels. The top-left plot (track 347) achieves a large L-measure because the first annotator's upper-level matches well to the second annotator's lower level, but not to the second annotator's upper-level. However, the two hierarchies are generally consistent with one another, and the L-measure identifies this consistency. The top-right plot (track 555) achieves large pairwise agreement at the upper level (aside from *E/E'*, these annotations are equivalent up to a permutation of the labels), but small pairwise agreement at the lower level, because the annotators disagree about whether the lower-level segment labels repeat in the second half of the song. Just as in the previous example (347), these two hierarchies are mutually consistent, and the L-measure produces a high score for this pair. The bottom-left plot (track 436) appears to consist of genuinely incompatible hierarchies, resulting in small scores across all metrics. The bottom-right plot (track 616) illustrates agreement in the upper level, but significant disagreement in the lower level, which is taken as evidence of hierarchical disagreement and produces a small L-measure (0.30).

**Figure 5:**
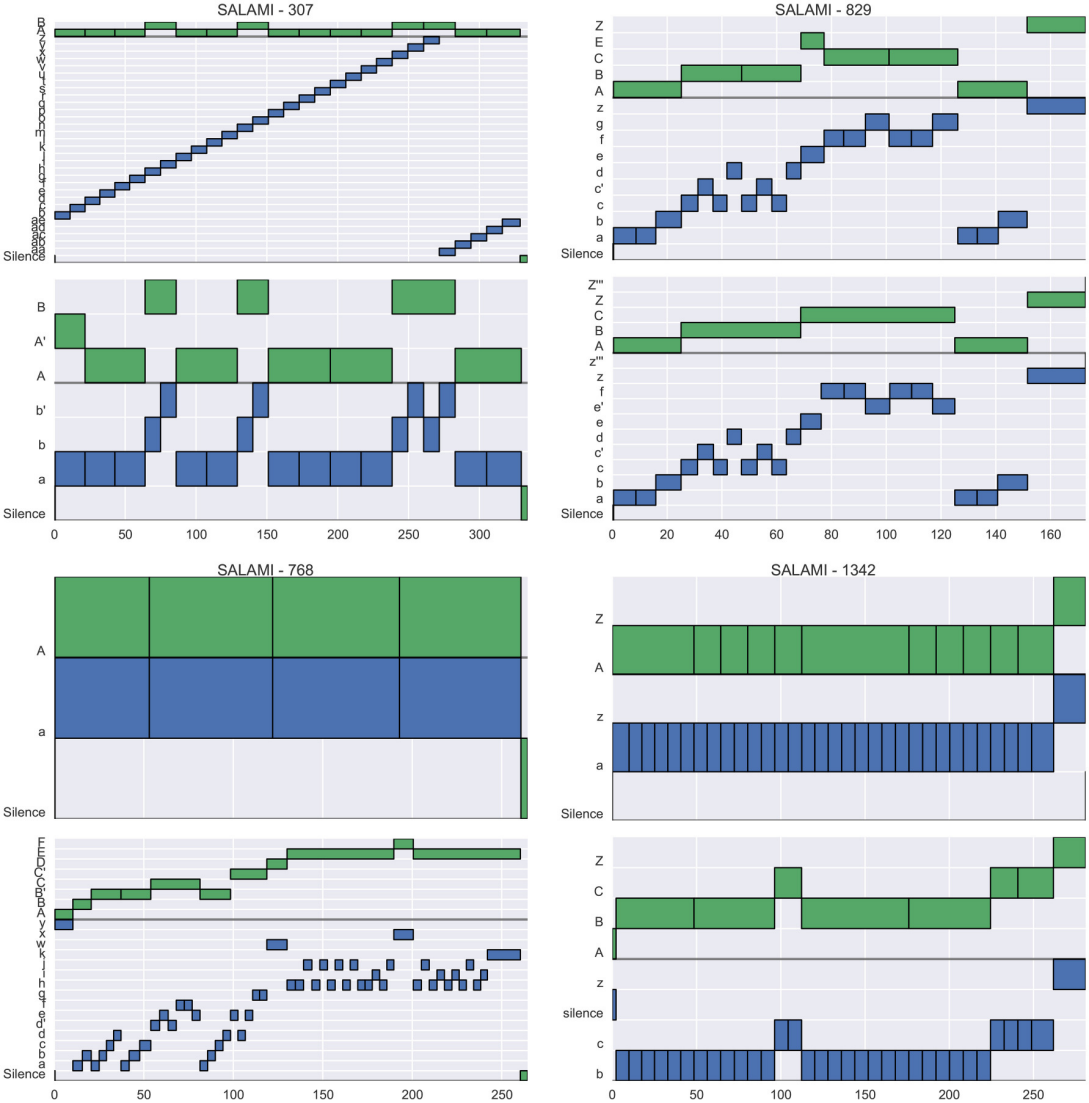
Four example tracks from SALAMI, one drawn from each quadrant of Figure 3 **(Right)**, which compares L-measure to the minimum of upper- and lower-level pairwise F-measure between tracks. **(Upper right)** Track 829 (*L* = 0.94, upper *F* = 0.93, lower *F* = 0.96) has high agreement at the both levels, and consequently a large L-measure. **(Upper left)** Track 307 (*L* = 0.94, upper *F* = 0.92, lower *F* = 0.11) has high agreement in the upper level, but the first annotator did not detect the same repetition structure as the second in the lower level. **(Bottom left)** Track 768 (*L* = 0.06, upper *F* = 0.43, lower *F* = 0.18) has little agreement at any level because the first annotator produced only single-label annotations. **(Bottom right)** Track 1342 (*L* = 0.39, upper *F* = 0.80, lower *F* = 0.80) has high pairwise agreement at both levels, but receives a small L-measure because the first annotator did not identify the distinct *C/c* sections indicated by the second annotator.

Similarly, Figure 5 illustrates examples drawn from each quadrant of the right plot in Figure 3 (across-layer minimum vs. L-measure). Here, the right column is of interest, since it lists annotations where the flat metrics agree at both levels (quadrants I and IV). The top-right plot (track 829) contains virtually identical hierarchies, and produces high scores under all metrics. The bottom-right plot (track 1342) consists of two essentially flat hierarchies where each lower-level contains the same label structure as the corresponding upper level. The large flat metrics here (*F* = 0.80) are easily understood since the majority of pairs of instants are labeled similarly in both annotations, excepting those (*u, v*) for which *u* is in section *C/c* for the second annotation and *v* is not, which are in the minority. The small L-measure (0.39) for this example is a consequence of the lack of label diversity in the first annotation, as compared to the second. By the definition in Equation (11), the L-measure only compares triples (*t, u, v*) where the labels for *u* and *v* differ, and in the second annotation, most of these triples contain an example from the *C/c* sections. Since the second annotation provides no information to disambiguate whether *C* is more similar to *A* or *Z*, the L-measure assigns a small score when compared to the first annotation.

A similar phenomenon can be observed in the bottom-left plot (track 768), in which the first annotator used a single label to describe the entire track in each level. In this case, nearly all of the comparison triples derived from the second annotation are not found in the first, resulting in an L-measure of 0.06. It is worth noting that the conditional entropy measures would behave similarly to the L-measure here, since the first annotation has almost no label entropy in either level.

To summarize, the L-measure broadly agrees with the level-by-level comparisons on the SALAMI dataset without requiring assumptions about equivalent level structure or performing comparisons between all pairs of levels. In the minority of cases (22%) where the L-measure substantially disagrees with the level-by-level comparison, the disagreements between metrics are often explained by the flat segmentations not accounting for hierarchical structure in the annotations. The exception to this are annotations with low label diversity across multiple levels, where the L-measure can assign a small score due to insufficiently many contrasting triples to form the evaluation (Figure 5, bottom-right).

## 5. Experiment 2: acoustic attributes

In the second experiment, we investigate annotator disagreement with respect to acoustic attributes. Two annotations that produce a small L-measure may be due to annotators responding to different perceptual or structural cues in the music.

### 5.1. Methods

**Figure 6:**
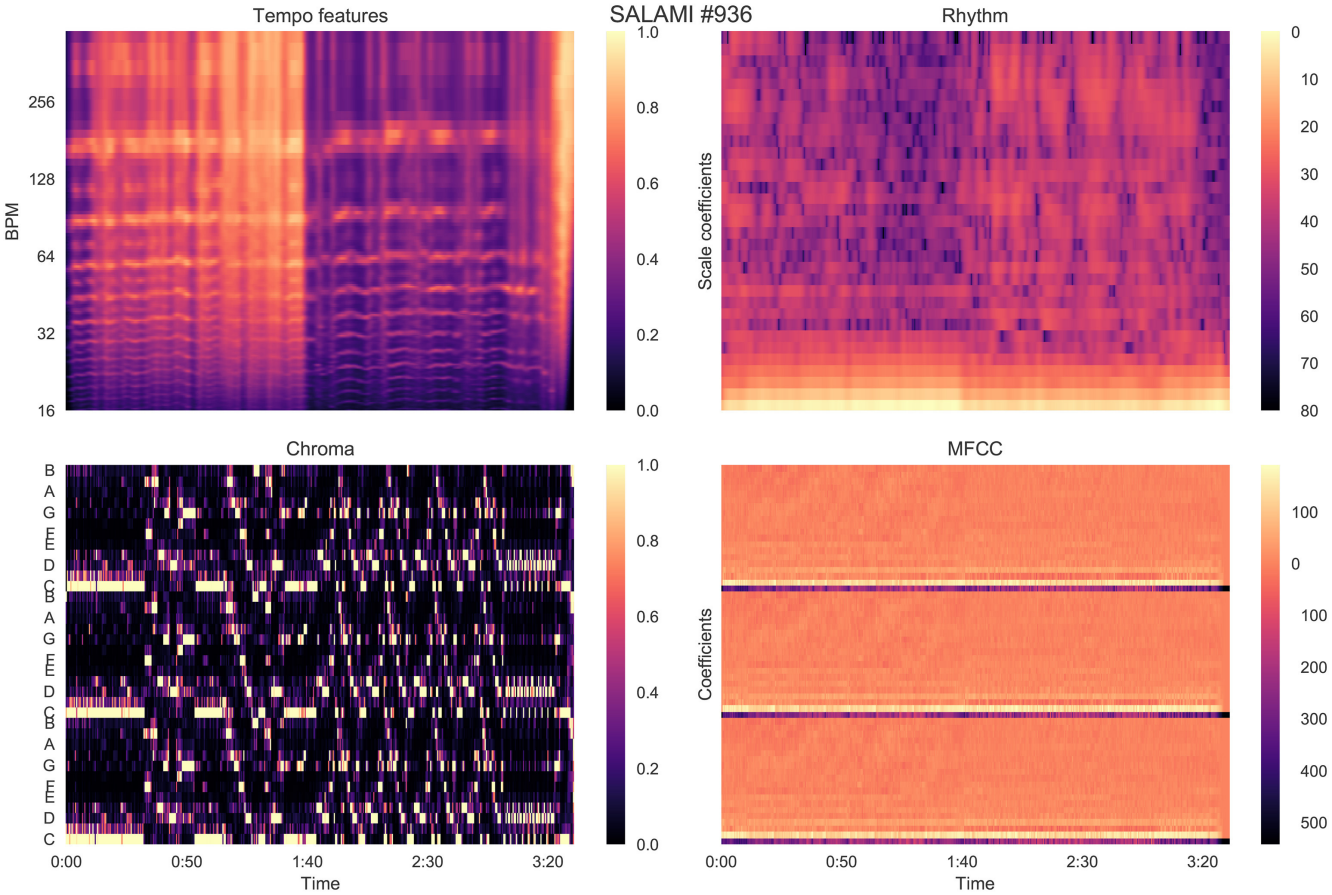
Features extracted from an example track in the SALAMI dataset, as described in Section 5.

To attempt to quantify attribute-based disagreement, we extracted four acoustic features from each recording, designed to capture aspects relating to tempo, rhythm, harmony, and timbre. Our hypothesis was that if hierarchical annotations receive small L-measure, and the annotators are indeed cued by different acoustic properties, then this effect should be evident when comparing annotations in a representation derived from acoustic features. All audio was down-sampled and mixed to 22,050 Hz mono prior to feature extraction, and all analysis was performed with librosa 0.5 dev (McFee et al., 2015b). A visualization of the features described in this section is provided in Figure 6.

#### 5.1.1. Tempo features

The tempo features consist of the short-time auto-correlation of the onset strength envelope of the recording. This feature loosely captures the timing structure of note onsets centered around each time point in the recording. The location of peaks in the onset strength auto-correlation can be used to infer the tempo at a given time.

The onset strength is computed by the spectral flux of a log-power Mel spectrogram of 128 bins sampled at a frame rate of ~ 43 Hz (hop size of 512 samples), and spanning the frequency range up to 11,025 Hz. The short-time auto-correlation is computed over centered windows of 384 frames (~ 8.9 s) using a Hann window, resulting in a feature matrix $X_{t}\in {\mathbb{R}}_{+}^{384\times T}$ (for *T* frames). The value at *X*_τ_ [*i, j*] is large if an onset envelope peak at frame *j* is likely to co-occur with another peak at frame *j* + *i*. Each column was normalized by its peak amplitude.

#### 5.1.2. Rhythm features

The rhythm features were computed by applying the scale (Mellin) transform to the tempo features derived above (Cohen, 1993; De Sena and Rocchesso, 2007). The scale transform magnitude has been used in prior work to produce an approximately tempo-invariant representation of rhythmic information (Holzapfel and Stylianou, 2011), so that similar rhythmic patterns played at different speeds result in similar feature representations.

At a high level, the scale transform works by re-sampling the onset auto-correlation—i.e., each column of *X*_τ_ defined above—on a logarithmic lag scale from a minimum lag *t*_0_ > 0 to the maximum lag, which in our case is the auto-correlation window length (384 frames). This transforms multiplicative scaling in time to an additive shift in logarithmic lag. The Fourier transform of this re-sampled signal then encodes additive shift as complex phase. Discarding the phase information, while retaining the magnitude, produces a tempo-invariant rhythm descriptor.

The scale transform has two parameters which must be set: the minimum lag *t*_0_ (in fractional frames), and the number of scale bins *n* (analogous to FFT bins), which we set to *t*_0_ = 0.5 and *n* = 64. Because the input (onset autocorrelation) is real-valued, its scale transform is conjugate-symmetric, so we discard the negative scale bins to produce a representation of dimension ⌊*n*/2⌋ + 1. The log-power of the scale transform magnitude was computed to produce the rhythm features $X_{\rho }\in {\mathbb{R}}^{33\times T}$.

#### 5.1.3. Chroma features

The harmony features were computed by extracting pitch class (*chroma*) features at the same time resolution as the tempo and rhythm features. Specifically, we applied the constant-Q transform magnitude using 36 bins per octave spanning the range (*C*1, *C*8), summed energy within pitch classes, and normalized each frame by peak amplitude. This resulted in a chromagram $X_{\chi }\in {\mathbb{R}}_{+}^{12\times T}$.

#### 5.1.4. Timbre features

Finally, timbre features were computed by extracting the first 20 Mel frequency cepstral coefficients (MFCCs) using a log-power Mel spectrogram of 128 bins, and the same frame rate as the previous features. This resulted in the MFCC feature matrix $X_{\mu }\in {\mathbb{R}}^{20\times T}$.

#### 5.1.5. Comparing audio to annotations

To compare audio features to hierarchical annotations, we converted the audio features described above to self-similarity matrices, described below. However, because the features are sampled at a high frame rate, the resulting *T* × *T* self-similarity matrices would require a large amount of memory to process (~ 3 GB for a four-minute song). We therefore down-sampled the feature matrices to a frame rate of 4 Hz by linear interpolation prior to computing the self-similarity matrices below. The tempo and rhythm features are relatively stable across large extents of time (each frame spans 8.9s), but the chroma and MFCC features are confined to much smaller local regions defined by their window sizes. To improve the stability of similarity for the chroma and MFCC features, each frame was extended by time-delay embedding (Kantz and Schreiber, 2004): concatenating the features of the previous two frames (after down-sampling). This provides a small amount of local context for each observation, and is a commonly used technique in music structure analysis algorithms (Serra et al., 2012).

We then computed self-similarity matrices for each feature with a Gaussian kernel:

(14) $$G[u,v]\;:={\text{e}}^{-\frac{1}{\sigma }\|X[u]-X[v]{\|}^{2}}$$

where *X*[*t*] denotes the feature vector at frame *t*, and the bandwidth σ is estimated as

(15) $$\sigma \;:={\text{mean}}_{u}\;{\text{median}}_{v}{\left\|X[u]-X[v]\right\|}^{2}.$$

Similarly, for each annotation, we computed the meet matrix *M* by Equation (10) (also at a frame rate of 4 Hz). Figures **9**, **10** illustrate examples of the feature-based self-similarity matrices, as well as the meet matrices for two annotations each.

To compare *M* to each of the feature-based self-similarity matrices *G*_τ_, *G*_ρ_, *G*_χ_, *G*_μ_, we first standardized each matrix by subtracting its mean value and normalizing to have unit Frobenius norm:

(16) $$\hat{D}\;:=\frac{D-{\text{mean}}_{u,v}D[u,v]}{{\left\|D-{\text{mean}}_{u,v}D[u,v]\right\|}_{\text{F}}}.$$

The inner product between normalized self-similarity matrices

(17) $${\langle \hat{M},\hat{G}\rangle }_{\text{F}}\;:=\sum_{u,v}\hat{M}[u,v]\hat{G}[u,v]$$

can be interpreted as a cross-correlation between the vectorized forms of *M* and *G*, and due to normalization, takes a value in [−1, 1]. Collecting these inner products against each *G* matrix results in a four-dimensional vector of feature-based similarity to the annotation *M*:

(18) $$z(M)\;:={\left({\langle \hat{M},{\hat{G}}_{i}\rangle }_{\text{F}}\right)}_{i\in \{\tau ,\rho ,\chi ,\mu \}}$$

To compare two annotations *H*^R^, *H*^E^ with meet matrices *M*^R^, *M*^E^, we could compute the Euclidean distance between the corresponding *z*-vectors. However, correlated features (such as tempo and rhythm) could artificially inflate the distance calculation. We therefore define a whitening transform *W*^−1^, where

(19) $$W[i,j]\;:={\langle {\hat{G}}_{i},{\hat{G}}_{j}\rangle }_{\text{F}}.$$

This provides a track-dependent, orthogonal basis for comparing meet matrices *M*^R^ and *M*^E^. The distance between annotations is then defined by

(20) $$\delta \left(H^{\text{R}},H^{\text{E}}\right)\;:=\sqrt{{\left(z\left(M^{\text{R}}\right)-z\left(M^{\text{E}}\right)\right)}^{\text{T}}W^{-1}\left(z\left(M^{\text{R}}\right)-z\left(M^{\text{E}}\right)\right)}.$$

By introducing the whitening transformation, we reduce the influence of correlations between acoustic features on the resulting annotation distance δ. A large distance δ indicates that the hierarchies correlate with different subsets of features, so we expect an inverse relationship between δ and the L-measure between the annotations.

### 5.2. Results and discussion

**Figure 7:**
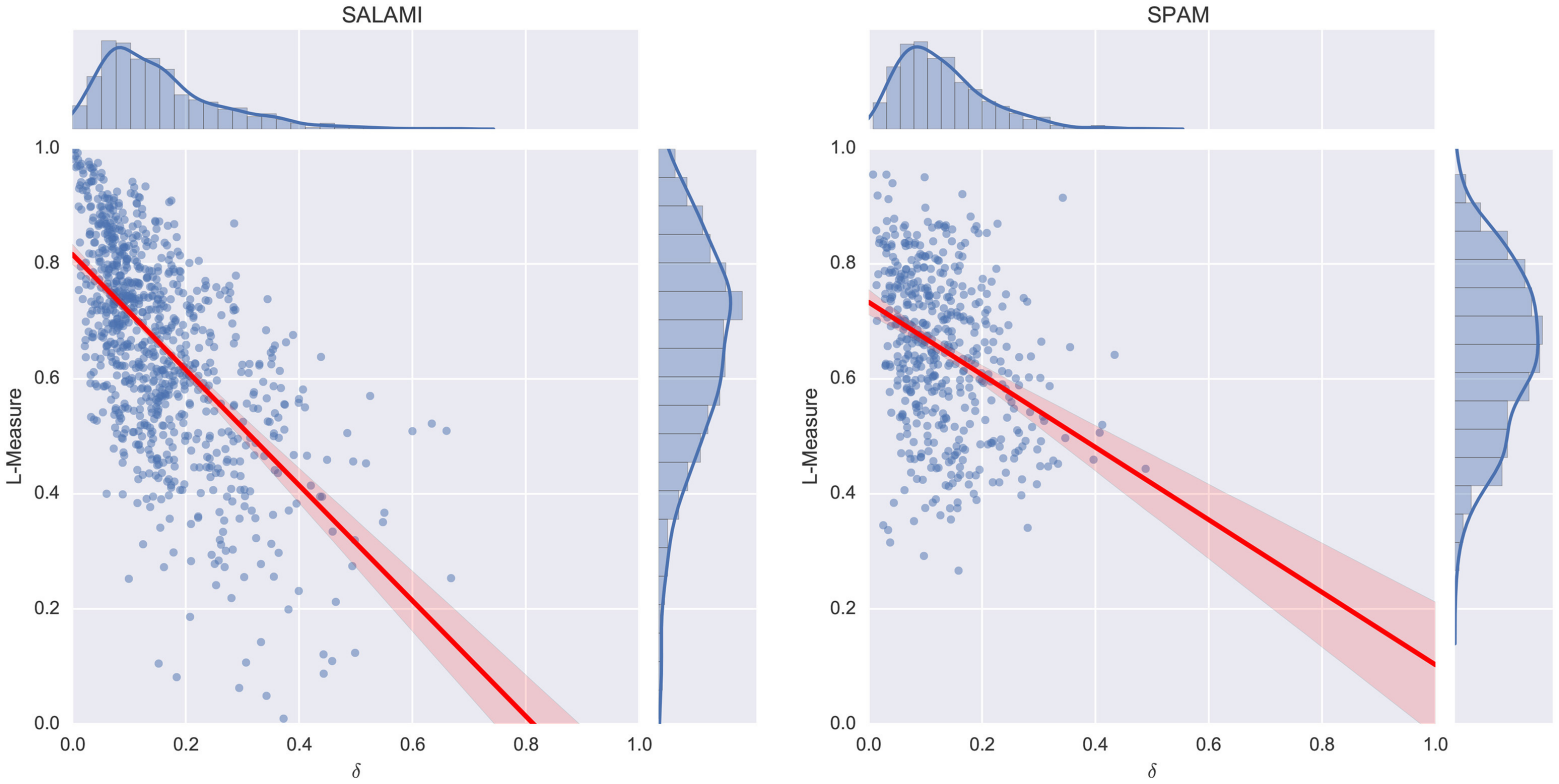
Feature correlation compared to L-measures on the SALAMI **(Left)** and SPAM **(Right)** datasets.

The results of the acoustic feature correlation experiment are displayed in Figure 7. As expected, the δ score is inversely related to the L-measure (*r* = −0.61 on the SALAMI data set, *r* = −0.32 on SPAM). Because the SPAM dataset was explicitly constructed from difficult examples, it produces smaller L-measures on average than the SALAMI dataset. However, the SPAM annotators did not appear to produce low label-diversity annotations that generate small L-measures, so the overall distribution is more concentrated. The δ distribution is similar across both datasets, which explains the apparently large discrepancy in correlation coefficients.

**Figure 8:**
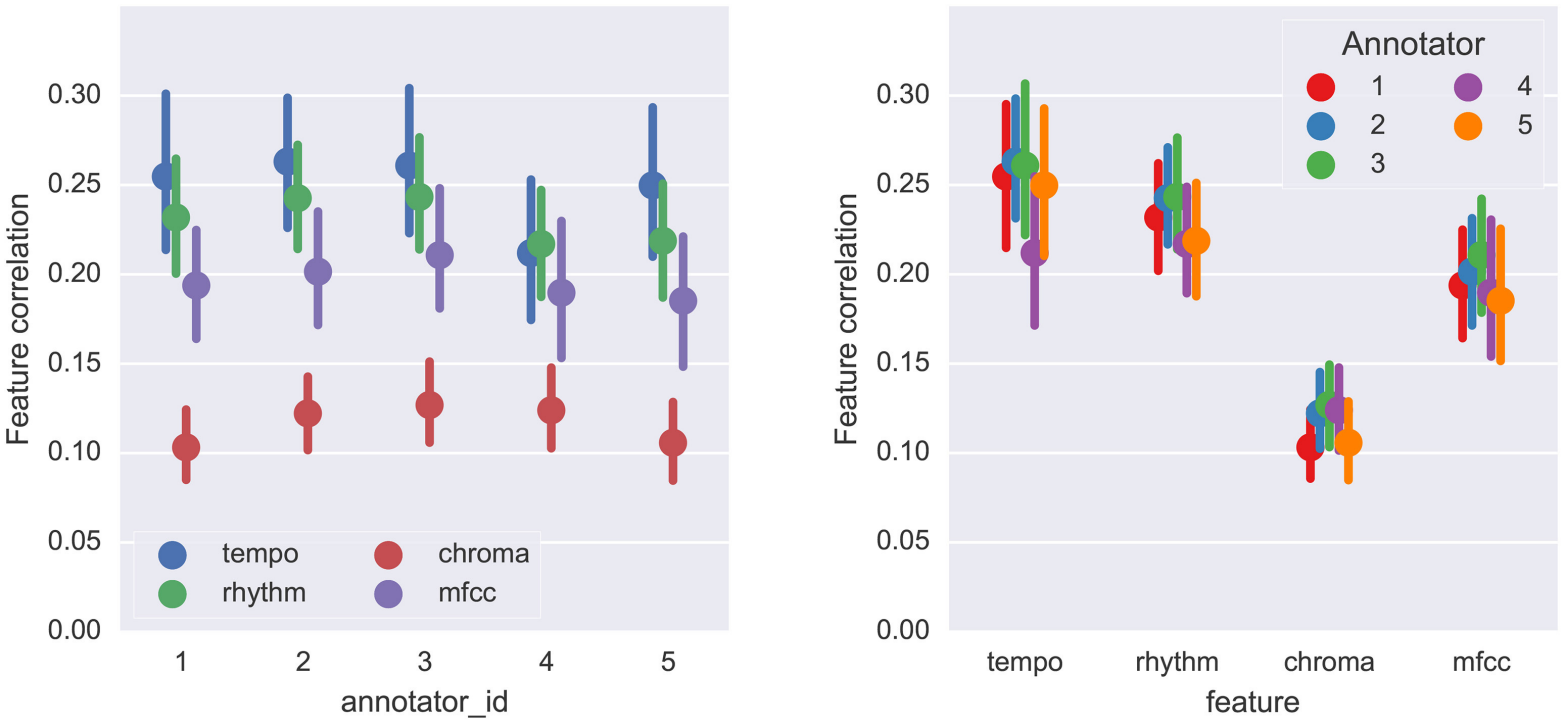
The mean feature correlation for each feature type and annotator on the SPAM dataset. Error bars indicate the 95% confidence intervals estimated by bootstrap sampling (*n* = 1, 000). **Left:** results are grouped by annotator ID; **Right:** results are grouped by feature type.

The estimated mean feature correlations are displayed in Figure 8. Because the SPAM dataset provides all combinations of the five annotators with the fifty tracks, it is more amenable to statistical analysis of annotator behavior than the SALAMI dataset. Using the SPAM dataset, we investigated the relationship between feature types and annotators. A two-way, repeated-measures ANOVA was performed with annotator and feature type as fixed effects and tracks as a random effect (all results Greenhouse-Geisser corrected). The main effects of annotator and feature type were both significant: *F*_(2.92, 142.85)_ = 3.44, *p* = 0.02, η^2^ = 0.068, $\eta_{p}^{2}=0.066$ for annotator and *F*_(2.52, 123.37)_ = 28.33, *p* = 1.49 × 10^−12^, η^2^ = 0.159, $\eta_{p}^{2}=0.366$ for feature type. The interaction effect was also significant, *F*_(8.26, 404.97)_ = 3.00, *p* = 2.46 × 10^−3^, η^2^ = 5.17 × 10^−3^, $\eta_{p}^{2}=0.058$. There was a large effect size for feature type and very small effect sizes for annotator and interaction.

Tukey's test for multiple comparisons revealed a significant difference between Annotators 3 and 4 (|*z*| = 2.88, *p* = 0.032) and a slight difference between 2 and 4 (|*z*| = 2.52, *p* = 0.086). Figure 8 (right) indicates that most of this difference is likely attributable to the tempo feature, which annotator 4 correlates with considerably less than the other annotators. These results demonstrate that a small set of annotators are likely to produce significantly different interpretations of musical structure, even when they are following a common set of guidelines.

**Figure 9:**
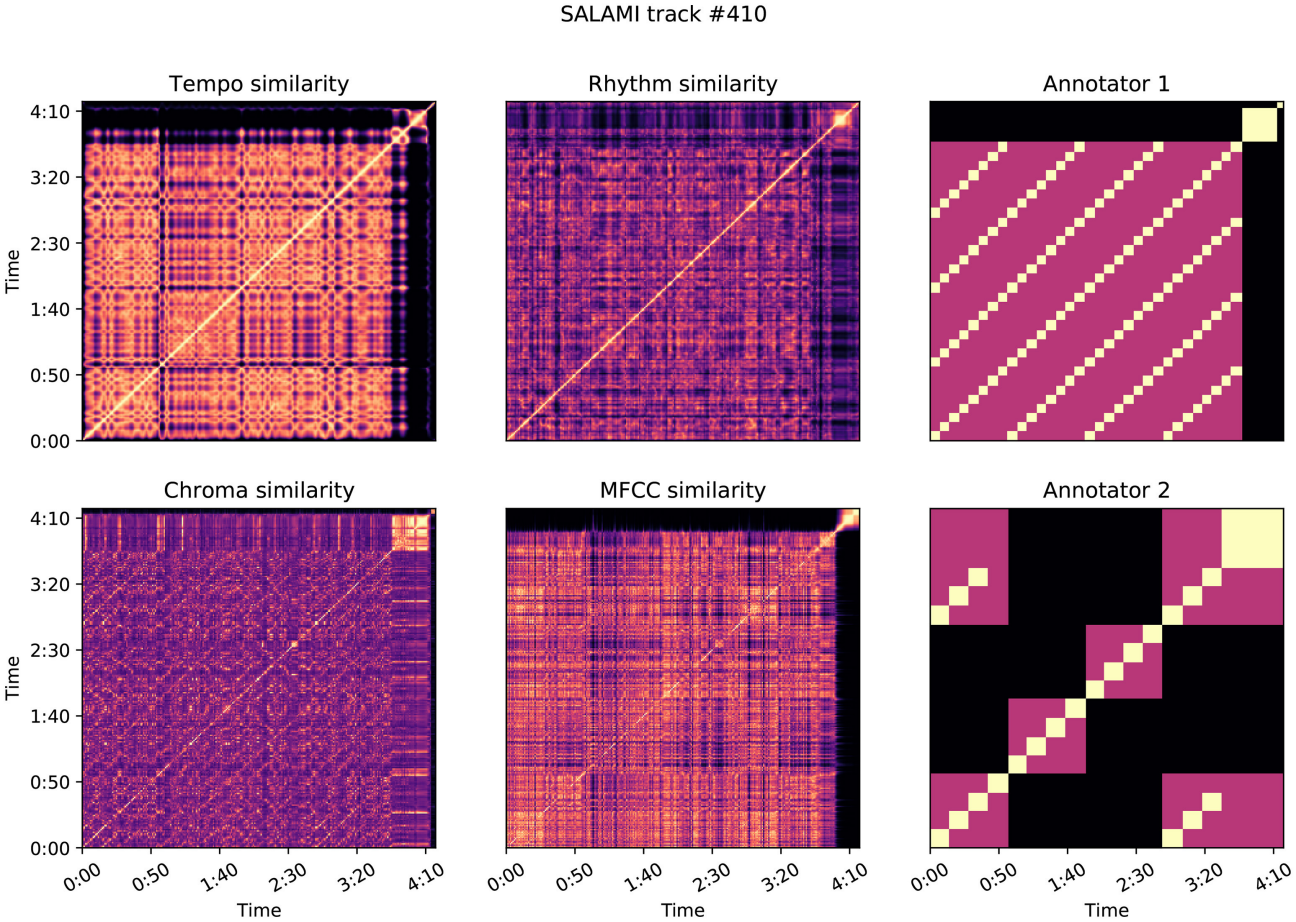
Feature correlation for SALAMI track #410: *Erik Truffaz*–*Betty*, which achieves δ = 0.67, L-measure = 0.25. The two annotations encode different hierarchical repetition structures, depicted in the meet matrices in the right-most column. Annotator 1's hierarchy is more highly correlated with the feature-based similarities: *z* = (0.62, 0.42, 0.26, 0.48) for tempo, rhythm, chroma, and MFCC, compared to *z* = (0.03, 0.07, 0.07, 0.04) for Annotator 2.

Figure 9 illustrates the self-similarity matrices for SALAMI track 410: *Erik Truffaz*–*Betty*, a jazz recording featuring trumpet, piano, bass, and drums. The two annotations for this track produce a small L-measure of 0.25, and a large δ score of 0.67. In this example, the two annotators appear to be expressing different opinions about the organization of the piece, as illustrated in the right-most column of Figure 9. Annotator 1 first separates the extended final fermata from the rest of the recording in the upper level, and then segments into repeated 4-bar progressions in the lower level. Annotator 2 groups by instrumentation or texture in the upper level, separating the piano and trumpet solos (center blocks) from the head section, and then grouping by repeated 8-bar segments. The first annotation correlates well with all of the feature-based similarity matrices, which exhibit low contrast for the majority of the piece. The second annotation is generally uncorrelated with the feature similarities, leading to the large δ score between the two. Note that this does not imply that one annotator was more “accurate” than the other, but it does suggest that the differences in the annotations can be attributed, at least in part, to perceptual characteristics of the music in question. In this case, Annotator 2 accounted for both instrumentation and harmony, while Annotator 1 accounted only for harmony.

**Figure 10:**
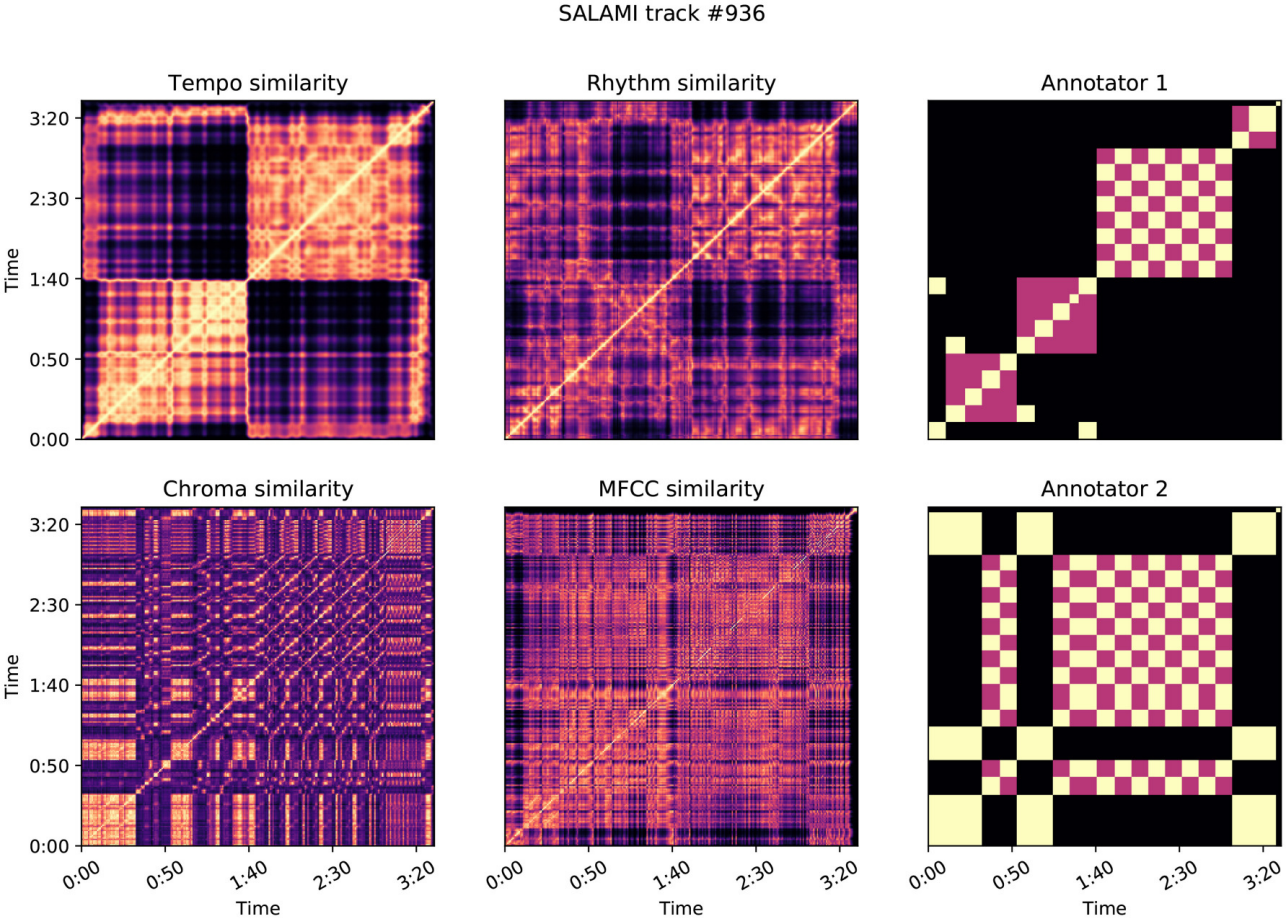
Feature correlation for SALAMI track #936: *Astor Piazzola*–*Tango Aspasionado*, which achieves δ = 0.45, L-measure = 0.46. Annotator 1 is highly correlated with the features: *z* = (0.57, 0.40, 0.11, 0.25) for tempo, rhythm, chroma, and MFCC, compared to *z* = (0.16, 0.12, 0.13, 0.25) for Annotator 2.

Figure 10 illustrates a second example, SALAMI track 936: *Astor Piazzola* – *Tango Aspasionado*, which produces L-measure of 0.46 and a relatively large δ = 0.45. The two annotators in this example have again identified substantially different large-scale structures, with the first annotation correlating highly with tempo (0.57) and rhythmic (0.40) similarity as compared to the second annotator (0.16 and 0.12, respectively). The second annotator identified repeating melodic and harmonic themes that persist across changes in instrumentation and rhythm. This persistence explains the comparatively low correlation scores for the tempo and rhythm features. The two annotators appear to disagree on the relative importance of rhythmic and instrumental characteristics, compared to melodic and harmonic features, in determining the structure of the piece.

In both of these examples, and as a general trend illustrated in Figure 8, annotations that relied on solely on harmony produced lower correlation scores than those which align with timbre and rhythm descriptors. This is likely a consequence of the dynamic structure of harmony and chroma representations, which evolve rapidly compared to the more locally stationary descriptors of timbre, rhythm, and tempo. Chroma self-similarity matrices (Figures 9, 10, bottom-left) tend to exhibit diagonal patterns rather than solid blocks of self-similar time intervals, which are easier to match against the annotation-based meet matrices (right column). It may be possible to engineer locally stable harmony representations that would be more amenable to this kind of correlation analysis, but doing so without supposing a pre-existing segmentation model is a non-trivial undertaking and beyond the scope of the present experiment.

## 6. Experiment 3: hierarchical algorithms

This last experiment focuses on using the L-measure to compare hierarchical results estimated by automatic approaches with those annotated by music experts. Assuming that the L-measure between human annotations defines the upper limit in terms of performance for the automated hierarchical segmentation task, we explore how the L-measure behaves when assessing this type of algorithms. We are particularly interested in better understanding how much room there is for improvement when designing new approaches to this task.

### 6.1. Methods

To the best of our knowledge, only two automatic methods that estimate hierarchical segmentations have been published with open source implementations: Laplacian structural decomposition (McFee and Ellis, 2014a), and Ordinal Linear Discriminant Analysis (McFee and Ellis, 2014b). The Laplacian method generates hierarchies of depth 10, where each layer *i* consists of *i* + 1 unique segment labels McFee and Ellis (2014a). For each layer index, this method first partitions the recording into a set of discontinuous clusters (segment labels), and then estimates segment boundaries according to changes in cluster membership between successive time instants. Consequently, each layer can have arbitrarily many segments, but the number of unique segment labels is always fixed.

The OLDA method, as described by McFee and Ellis (2014b), operates by agglomerative clustering of time instants into segments, resulting in a binary tree with time instants at the leaves, and the entire recording at the root. Each layer *i* of this tree has *i* + 1 contiguous segments, and the tree is automatically pruned based on the statistics of segment lengths and the overall track duration. This results in a hierarchy of variable depth, typically between 15 and 30 levels, where each level can be seen as splitting one segment from the previous level into two. Because OLDA only estimates segment boundaries, segment labels were estimated at each level by using the 2D-Fourier Magnitude Coefficients method (Nieto and Bello, 2014), which yields state-of-the-art results in terms of automatic flat segment label prediction. The 2D-FMC method is set to identify a maximum of 7 unique labels per level of segmentation, as this number was previously found to produce the best results in The Beatles^8^ and SALAMI datasets. These sets are the most popular in the task of structural segmentation, and it is a standard practice to tune the parameters according to them (Kaiser and Sikora, 2010; Nieto and Jehan, 2013; Nieto and Bello, 2014).

The standard approach to measuring the performance of automatic algorithms is to compare the average scores derived from a sample of tracks, each of which has one “ground truth” annotation. However, as demonstrated in the previous sections, there is still significant disagreement between annotators when it comes to hierarchical segmentation, so selecting a single annotation to use as a point of reference would bias the results of the evaluation. Instead, we compared the output of each algorithm to all annotations for a given track, with results presented in terms of the full empirical distribution over scores rather than the mean score. We quantify the difference in distributions by the two-sample Kolmogorov-Smirnov statistic, which measures the maximum difference between the empirical cumulative distributions: a small value (near 0) indicates high similarity, a large value (near 1) indicates low similarity. For this experiment, the set of human annotations had a privileged interpretation (compared to the automatic methods), so we reported L-precision, L-recall, and L-measure separately.

Both algorithms (OLDA and Laplacian) were run on both datasets (SALAMI and SPAM) using the open-source implementations found in the Music Structure Analysis Framework, version 0.1.2-dev (Nieto and Bello, 2016). All algorithm parameters were left at their default values.

### 6.2. Results and discussion

**Figure 11:**
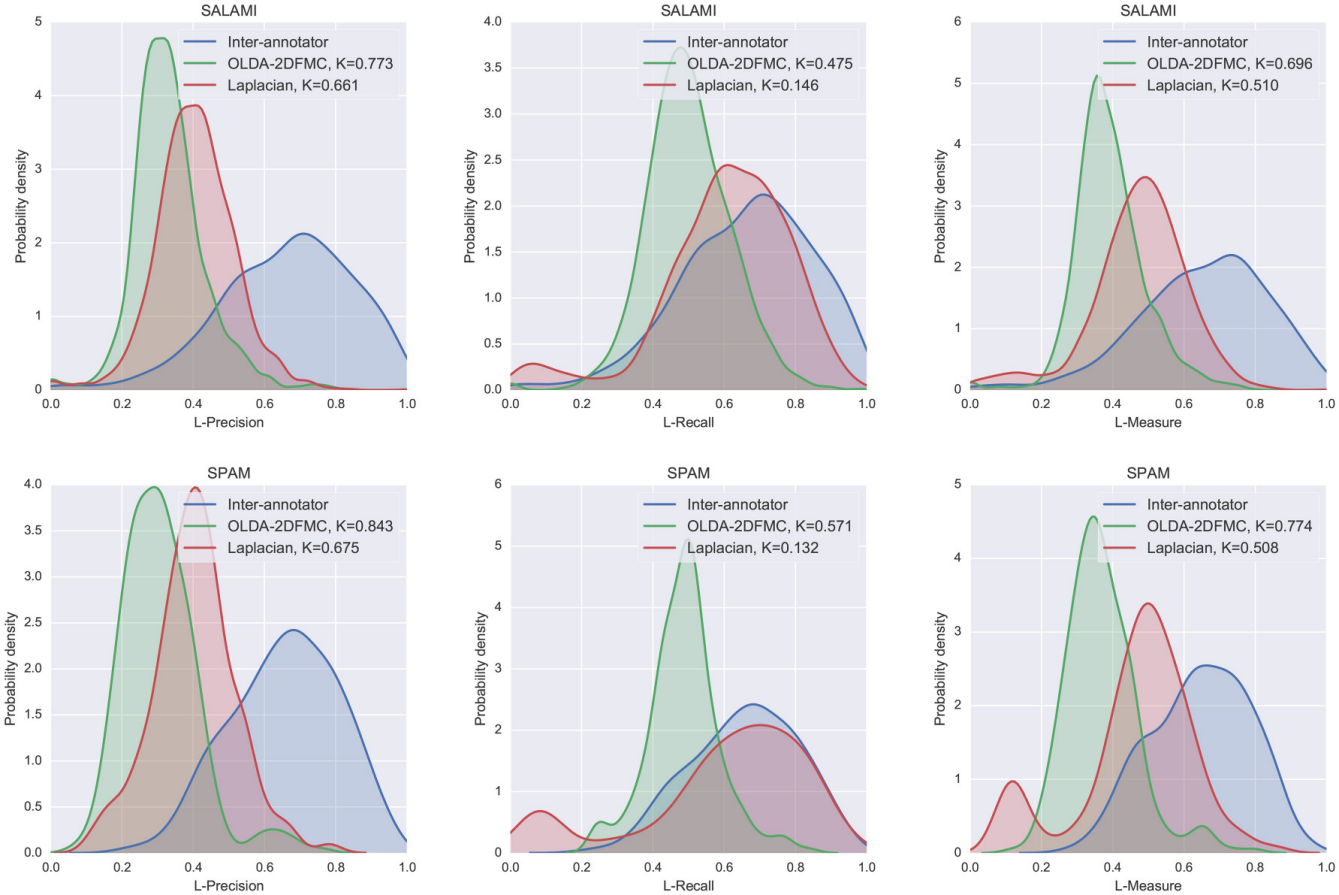
The distribution L-measure scores for inter-annotator agreement, OLDA-2DFMC, and Laplacian on the SALAMI **(Top row)** and SPAM **(Bottom row)** datasets. The left, middle, and right columns compare algorithm L-precision, L-recall, and L-measure to inter-annotator scores. For each algorithm, the two-sample Kolmogorov-Smirnov test statistic *K* is computed against the inter-annotator distribution (smaller *K* is better).

The results of the automatic hierarchical segmentation algorithm experiment are displayed in Figure 11. Both algorithms achieve larger average L-recall (center column) than L-precision (left column), which suggests that the automated methods, which produce much deeper hierarchies than the reference annotations, have identified more detailed structures than were encoded by the human annotators. Notably, the Laplacian method achieved a recall distribution quite close to that of the human annotators. This indicates that the L-measure is robust to differences in hierarchical depth: structures encoded in the depth-2 human annotations can also be found in the depth-10 automatic annotations.

The right column shows the total L-measure distribution (combining precision and recall). In both datasets, the Laplacian method was significantly more similar to the inter-annotator distribution than the OLDA-2DFMC method was, despite the mode at the bottom of the L-measure scale visible in Figure 11 (right). The region of low performance can be attributed to an apparent weakness of the method on longer recordings (e.g., *SALAMI-478* at 525 s, or *SALAMI-108* at 432 s) where it tends to over-emphasize short discontinuities and otherwise label the remainder of the track as belonging primarily to one component. This behavior can also be seen in the SALAMI distribution, though such examples make up a smaller portion of the corpus, and therefore exert less influence on the resulting distribution.

The results of this experiment demonstrate a rather large gap between the distribution of inter-annotator agreement and algorithm-annotator agreement. In the examples presented here, and especially the Laplacian method, much of this gap can be attributed to low precision. Low precision may arise naturally from comparisons between deep and shallow hierarchies. Because the reference annotations in both SALAMI and SPAM have fixed depth, this effect is not observable in the inter-annotator comparison distribution. This effect suggests a trade-off between precision and recall as a function of hierarchy depth. If a practitioner was interested in bounding hierarchy depth to optimize this trade-off, the L-measure would provide a means to do so.

## 7. General discussion

From the perspective of music informatics research, the hierarchical evaluation technique described here opens up new possibilities for algorithm development. Most existing automatic segmentation methods, in one way or another, seek to optimize the existing metrics for flat boundary detection and segment label agreement. Boundary detection is often modeled as a binary classification problem (boundary/not-boundary), and labeling is often modeled as a clustering problem. The L-measure suggests instead to treat both problems from the perspective of similarity ranking, and could therefore be used to define an objective function for a machine-learning approach to hierarchical segmentation.

As demonstrated in Section 4, the L-measure can reduce bias in the evaluation due to superficial differences between two hierarchical segmentations, which better exposes meaningful structural discrepancies. Still, there appears to be a considerable amount of inter-annotator disagreement in commonly used corpora. Disagreement is a pervasive problem in music informatics research, where practitioners typically evaluate an algorithm by comparing its output to a single “ground truth” annotation for each track in the corpus. The evaluation described in Section 6 represents a potentially viable alternative method of evaluation, which seeks not to measure “agreement” against human annotators, but rather to match the distribution of agreement *between* human annotators. This approach could be easily adapted to other tasks involving high degrees of inter-annotator disagreement, such as chord recognition or automatic tagging.

While the L-measure resolves some problems with evaluating segmentations across different levels, it still shares some limitations with previous label-based evaluation metrics. Notably, none of the existing methods can distinguish between adjacent repetitions of the same segment label (*aa*) from a single segment spanning the same time interval (*A*). This results in an evaluation which is blind to boundaries between similarly labeled segments, and therefore discards important cues indicating repetition. Similarly, variation segments—e.g., (*A, A'*) in SALAMI notation—are always treated as distinct, and equally distinct as any other pair of dissimilar segments (*A*,*B*). While the L-measure itself does not present a solution to these problems, its ability to support hierarchies of arbitrary depth could facilitate solutions in the future. Specifically, one could augment an existing segmentation with additional lower layers that distinguish among each instance of a label, so that *a, a* decomposes into *a1, a2*, without losing the information that both segments ultimately receive the same label. Similarly, variations could be resolved by introducing a layer above which unifies *A, A'* both as of type *A*. Because this approach requires significant manipulation of annotations, we leave it as future work to investigate its effects.

The work described here also offers both insight and a potential tool for researchers in the field of music cognition. The results from Experiment 1 reveal that flat segmentation metrics are confounded by superficial differences between otherwise consistent hierarchical annotations, while the L-measure is robust to these differences. The L-measure can therefore provide a window into the individual differences inherent in the perception of musical structure. Furthermore, the L-measure can provide a quantitative metric for directly comparing hierarchical analyses of musical form in experimental work. It can serve as a means to objectively assess response similarity between subjects on tasks that require analysis of metrical, grouping, and prolongational hierarchies.

The results of Experiment 2 present evidence for distinct modes of listening predicated on different acoustical features of the music. Comparing differences in feature correlations can help identify potential causal factors contributing to listener interpretation of musical form. The feature analysis offers objective evidence in support of qualitative observations for how and why listeners interpret musical structure differently, particularly in cases of significant disagreement.

## Author contributions

All authors contributed to the research conceptually, including the experimental design and data interpretation. All authors also contributed to writing and editing the paper. Additional individual contributions are as follows: BM contributed to data preparation, software implementation, and conducted experiments; ON contributed to data preparation and conducted experiments; MF contributed to part of the statistical analysis.

### Conflict of interest statement

The authors declare that the research was conducted in the absence of any commercial or financial relationships that could be construed as a potential conflict of interest.

## Footnotes

^1^In the context of the present article, these two elements (cultural and universal) are not differentiated because the listeners who provide hierarchical analyses all had prior experience with Western music.

^2^The SALAMI annotation guide is available at http://music.mcgill.ca/~jordan/salami/SALAMI-Annotator-Guide.pdf.

^3^https://github.com/DDMAL/salami-data-public/pull/15

^4^Although segmentations are typically produced by annotators without reference to a fixed time grid, it is standard to evaluate segmentations after re-sampling segment labels at a standard resolution of 10 Hz (Raffel et al., 2014), which we adopt for the remainder of this article.

^5^It has been recently noted that maximum-entropy normalization can artificially inflate scores in practice because the marginal distribution **P**^E^ is often far from uniform. See https://github.com/craffel/mir_eval/issues/226 for details. For the remainder of this article, we focus comparisons on the pairwise classification metrics, but include NCE scores for completeness.

^6^If *S*_0_ is not provided, it can be trivially synthesized. Including *S*_0_ in the hierarchy is useful for ensuring that the metrics derived in Section 3.2.1 are well-formed.

^7^Our implementations for the experiments included in this paper are available at https://github.com/bmcfee/segment_hierarchy_labels.

^8^http://isophonics.net/content/reference-annotations-beatles

## References

[B1] Balke S. Arifi-Müller V. Lamprecht L. Müller M. (2016). Retrieving audio recordings using musical themes, in Proceedings of the IEEE International Conference on Acoustics, Speech, and Signal Processing (ICASSP) (Shanghai).

[B2] Barwick L. (1989). Creative (ir) regularities: the intermeshing of text and melody in performance of central australian song. Aus. Aboriginal Stud. 1, 12–28.

[B3] Bharucha J. J. Curtis M. Paroo K. (2006). Varieties of musical experience. Cognition 100, 131–172. 10.1016/j.cognition.2005.11.008 16412410

[B4] Bruderer M. J. (2008). Perception and Modeling of Segment Boundaries in Popular Music. PhD thesis, Doctoral dissertation, JF Schouten School for User-System Interaction Research, Technische Universiteit Eindhoven.

[B5] Clayton M. (1997). Le mètre et le tāl dans la musique de l'inde du nord. Cahiers Musiques Traditionnelles 10, 169–189. 10.2307/40240271

[B6] Cohen L. (1993). The scale representation. IEEE Trans. Signal Process. 41, 3275–3292. 10.1109/78.258073

[B7] Cook N. (2003). Music as performance, in The Cultural Study of Music A Critical Introduction, eds Clayton M. Herbert T. Middleton R. (New York, NY: Routledge), 204–214.

[B8] Davies M. E. P. Hamel P. Yoshii K. Goto M. (2014). Automashupper: automatic creation of multi-song music mashups. IEEE/ACM Trans. Audio Speech Lang. Process. 22, 1726–1737. 10.1109/TASLP.2014.2347135

[B9] De Sena A. Rocchesso D. (2007). A fast mellin and scale transform. EURASIP J. Appl. Signal Process 2007, 75–84.

[B10] Deutsch D. (ed.). (1999). Grouping mechanisms in music, in The Psychology of Music, 2nd Edn. (New York, NY: Academic Press), 299–348. 10.1016/B978-012213564-4/50010-X

[B11] Deutsch D. Feroe J. (1981). The internal representation of pitch sequences in tonal music. Psychol. Rev. 88, 503–522. 10.1037/0033-295X.88.6.503

[B12] Drake C. (1998). Psychological processes involved in the temporal organization of complex auditory sequences: universal and acquired processes. Music Percept. Interdisc. J. 16, 11–26. 10.2307/40285774

[B13] Drake C. El Heni J. B. (2003). Synchronizing with music: intercultural differences. Anna. N.Y. Acad. Sci. 999, 429–437. 10.1196/annals.1284.053 14681167

[B14] Farbood M. M. Heeger D. J. Marcus G. Hasson U. Lerner Y. (2015). The neural processing of hierarchical structure in music and speech at different timescales. Front. Neurosci. 9:157. 10.3389/fnins.2015.00157 26029037 PMC4429236

[B15] Grill T. Schlüter J. (2015). Music boundary detection using neural networks on combined features and two-level annotations, in Proceedings of the 16th International Society for Music Information Retrieval Conference (Málaga: Citeseer).

[B16] Herremans D. Chew E. (2016). Music Generation with Structural Constraints: An Operations Research Approach. Louvain-La-Neuve.

[B17] Holzapfel A. Stylianou Y. (2011). Scale transform in rhythmic similarity of music. IEEE Trans. Audio Speech Lang. Process. 19, 176–185. 10.1109/TASL.2010.2045782

[B18] Kaiser F. Sikora T. (2010). Music structure discovery in popular music using non-negative matrix Factorization, in Proceedings of the 11th International Society of Music Information Retrieval (Utrecht), 429–434.

[B19] Kantz H. Schreiber T. (2004). Nonlinear Time Series Analysis, Vol. 7. Cambridge, UK: Cambridge University Press.

[B20] Krumhansl C. L. Jusczyk P. W. (1990). Infants' perception of phrase structure in music. Psychol. Sci. 1, 70–73. 10.1111/j.1467-9280.1990.tb00070.x

[B21] Lerdahl F. (1988). Tonal pitch space. Music Percept. 5, 315–349. 10.2307/40285402

[B22] Lerdahl F. Jackendoff R. (1983). An overview of hierarchical structure in music. Music Percept. Interdisc. J. 1, 229–252. 10.2307/40285257

[B23] Levy M. Sandler M. (2008). Structural segmentation of musical audio by constrained clustering. IEEE Trans. Audio Speech Lang. Process. 16, 318–326. 10.1109/TASL.2007.910781

[B24] Lukashevich H. (2008). Towards Quantitative Measures of Evaluating Song Segmentation, in Proceedings of the 10th International Society of Music Information Retrieval (Philadelphia, PA), 375–380.

[B25] McAdams S. (1989). Psychological constraints on form-bearing dimensions in music. Contemp. Music Rev. 4, 181–198. 10.1080/07494468900640281

[B26] McFee B. Ellis D. P. W. (2014a). Analyzing song structure with spectral clustering, in Proceedings of the 15th International Society for Music Information Retrieval Conference (Taipei), 405–410.

[B27] McFee B. Ellis D. P. W. (2014b). Learning to Segment Songs With Ordinal Linear Discriminant Analysis, in Proceeings of the 39th IEEE International Conference on Acoustics Speech and Signal Processing (Florence), 5197–5201.

[B28] McFee B. Nieto O. Bello J. (2015a). Hierarchical evaluation of segment boundary detection, in 16th International Society for Music Information Retrieval Conference (ISMIR) (Malaga).

[B29] McFee B. Raffel C. Liang D. Ellis D. P. W. McVicar M. Battenberg E. (2015b). Librosa: audio and music signal analysis in pyhon, in Proceeding of the 14th Python in Science Conference (Austin, TX), 18–25.

[B30] Nan Y. Knösche T. R. Friederici A. D. (2006). The perception of musical phrase structure: a cross-cultural ERP study. Brain Res. 1094, 179–191. 10.1016/j.brainres.2006.03.115 16712816

[B31] Nieto O. (2015). Discovering Structure in Music: Automatic Approaches and Perceptual Evaluations. Ph.d dissertation, New York University.

[B32] Nieto O. Bello J. P. (2014). Music segment similarity using 2D-Fourier magnitude coefficients, in Proceedings of the 39th IEEE International Conference on Acoustics Speech and Signal Processing (Florence), 664–668.

[B33] Nieto O. Bello J. P. (2016). Systematic exploration of computational music structure research, in Proceedings of ISMIR (New York, NY).

[B34] Nieto O. Farbood M. M. Jehan T. Bello J. P. (2014). Perceptual analysis of the f-measure for evaluating section boundaries in music, in Proceedings of the 15th International Society for Music Information Retrieval Conference (ISMIR 2014) (Taipei), 265–270.

[B35] Nieto O. Jehan T. (2013). Convex non-negative matrix factorization for automatic music structure identification, in Proceedings of the 38th IEEE International Conference on Acoustics Speech and Signal Processing (Vancouver, BC), 236–240.

[B36] Paulus J. Müller M. Klapuri A. (2010). State of the art report: audio-based music structure analysis, in ISMIR (Utrecht), 625–636.

[B37] Raffel C. McFee B. Humphrey E. J. Salamon J. Nieto O. Liang D. (2014). mir_eval: a transparent implementation of common mir metrics, in Proceedings of the 15th International Society for Music Information Retrieval Conference, ISMIR (Taipei: Citeseer).

[B38] Roy P. Perez G. Rgin J.-C. Papadopoulos A. Pachet F. Marchini M. (2016). Enforcing structure on temporal sequences: the allen constraint, in Proceedings of the 22nd International Conference on Principles and Practice of Constraint Programming - CP (Toulouse: Springer).

[B39] Serra J. Müller M. Grosche P. Arcos J. L. (2012). Unsupervised detection of music boundaries by time series structure features, in Twenty-Sixth AAAI Conference on Artificial Intelligence (Toronto, ON).

[B40] Shaffer L. H. Todd N. (1987). The interpretive component in musical performance, in Action and Perception in Rhythm and Music, ed Gabrielsson A. (Stockholm: Royal Swedish Academy of Music), 139–152.

[B41] Smith J. B. Burgoyne J. A. Fujinaga I. De Roure D. Downie J. S. (2011). Design and creation of a large-scale database of structural annotations, in ISMIR, Vol. 11 (Miami, FL), 555–560.

[B42] Smith J. B. Chuan C.-H. Chew E. (2014). Audio properties of perceived boundaries in music. IEEE Trans. Multimedia 16, 1219–1228. 10.1109/TMM.2014.2310706

[B43] Todd N. (1985). A model of expressive timing in tonal music. Music Percept. 3, 33–57. 10.2307/40285321

[B44] Trehub S. E. Hannon E. E. (2006). Infant music perception: domain-general or domain-specific mechanisms? Cognition 100, 73–99. 10.1016/j.cognition.2005.11.006 16380107

[B45] Weiß C. Arifi-Müller V. Prätzlich T. Kleinertz R. Müller M. (2016). Analyzing measure annotations for western classical music recordings, in Proceedings of the 17th International Society for Music Information Retrieval Conference (New York, NY).

